# Ocular Microbiome in Dupilumab‐Induced Ocular Surface Disease

**DOI:** 10.1111/all.70104

**Published:** 2025-10-16

**Authors:** Andrea Leonardi, Riccardo Frizzo, Fabiano Cavarzeran, Jerome Ozkan, Umberto Rosani

**Affiliations:** ^1^ Department of Neuroscience, Ophthalmology Unit University of Padova Padova Italy; ^2^ Department of Biology University of Padova Padova Italy; ^3^ School of Optometry and Vision Science, Faculty of Medicine and Health, School of Biological, Earth and Environmental Sciences, Faculty of Science University of New South Wales Sydney New South Wales Australia

**Keywords:** atopic dermatitis, biologics, microbiome, ocular allergy

To the Editor,

Patients with moderate–severe atopic dermatitis (AD) receive a great benefit from the use of dupilumab, a human IgG4 monoclonal antibody inhibiting IL‐4/IL‐13 signaling. However, randomized controlled trials and real‐world studies showed, in 30%–50% of cases, the development of dupilumab‐induced ocular surface disease (DIOSD), characterized by blepharitis, conjunctivitis, dry eye disease (DED), limbal inflammation, evolving into scarring, ectropion, and keratitis [1]. Higher risk of DIOSD has been associated with AD severity, history of eye disease, high serum IgE levels, and eosinophilia [1]. Metabolic differences between AD patients with and without DIOSD have also been reported [2]. Similarities between DIOSD and DED were suggested because of the decreased number of conjunctival goblet cells and secreted mucins in both [1]. Recently, a shift from a mixed Th2/Th17 toward a Th1/Th17 cytokine profile was shown in AD patients developing DIOSD with a decreased richness of the ocular microbiome [3]. In our real‐world cohort of 32 dupilumab‐treated AD patients, 17 (53%) had no adverse effects, and 15 (47%) were affected by DIOSD. We conducted a pilot study in 13 dupilumab‐treated AD patients, 5 without ocular symptoms (No‐DIOSD) and 8 with ocular side effects (DIOSD), and in 6 healthy subjects (CT) applying full‐length 16S rRNA gene amplicon sequencing (Oxford Nanopore Technologies, ONT). After recording signs and symptoms (Table S1), each subject was swabbed at the inferior fornix of the right eye, without topical anesthetics, using a single‐use, dry, sterile nylon swab (eSwab, Copan Diagnostics Inc., Murrieta, CA, USA), placed in sterile vials and stored at −20°C. The study adhered to the tenets of the Helsinki Declaration and was approved by the Institutional Review Board. Written informed consent was obtained from all patients/subjects enrolled. DNA was extracted using the NucleoSpin Tissue XS KIT (Machery‐Nagel, Düren, Germany) and used as a template to amplify 16S rRNA genes, prepare, and sequence one multiplexed ONT library. Since the quantity of DNA per patient was not sufficient for individual resolution, samples were pooled into 3 groups, CT, No‐DIOSD, and DIOSD with total DNA mass of 3.2, 2.1, and 1.6 ng, respectively. We clustered OTUs (similarity threshold 95% over 80% of sequence length) from the 27,173 sequencing reads, and we taxonomically assigned them at the genus level (Table 1, Figure 1). Six phyla were recognized in the DIOSD, 9 in the No‐DIOSD, and 15 in the CT group (Table S2). The top 5 genera were *Moraxella*, *Paracoccus*, *Streptococcus, Cutibacterium*, and *Staphylococcus* in CT; *Moraxella, Paracoccus*, *Massilia*, *Comamonas*, and *Acinetobacter* in No‐DIOSD; *Moraxella*, *Streptococcus, Paracoccus*, *Acinetobacter*, and *Staphylococcus* in DIOSD (Figure 1; Table S3). Although we can't identify which species prevail over the others between the No‐DIOSD and DIOSD patients, we showed some difference in microbiota composition between the 3 groups. Similar to previous studies [4], *Actinomycetota* (*Actinobacteria*), *Bacillota* (*Firmicutes*), and *Pseudomonatota* (*Proteobacteria*) were the most prevalent phyla in the CT conjunctiva, with evidence of local dysbiosis in AD patients, either with or without DIOSD, both at the phylum and genus levels. A reduced ocular microbial diversity and an increase in the relative abundance of pathogenic bacteria was similarly demonstrated in DED [5], suggesting that higher microbial ocular diversity may help in maintaining ocular surface homeostasis [6]. Alterations in the composition and structure of the ocular microbiota in AD patients may disrupt the local microenvironment, triggering inflammatory responses that may be exacerbated by dupilumab treatment. The main limitation in our study was the difficulty in obtaining full‐length 16S rRNA gene amplicons from our patients, probably due to the lower microbial load in ocular samples [4]. A longitudinal sampling protocol may have revealed microbiome changes due to therapeutic strategies for DIOSD. Further investigation is needed to highlight any pathogenic functions related to the specific microbe and the development of DIOSD, as well as to further develop the analysis based on full‐length 16S rRNA gene amplicon sequencing starting from ocular samples.

**TABLE 1:** Total read counts and number of genera in normal subjects (CT), dupilumab‐treated patients without ocular side effects (No‐DIOSD), and dupilumab‐treated patients with ocular side effects (DIOSD).

	CT	No‐DIOSD	DIOSD
Total read counts	10,906	6821	9446
Number of genera	136	59	50
Number of unique phyla	15	9	6

**FIGURE 1:**
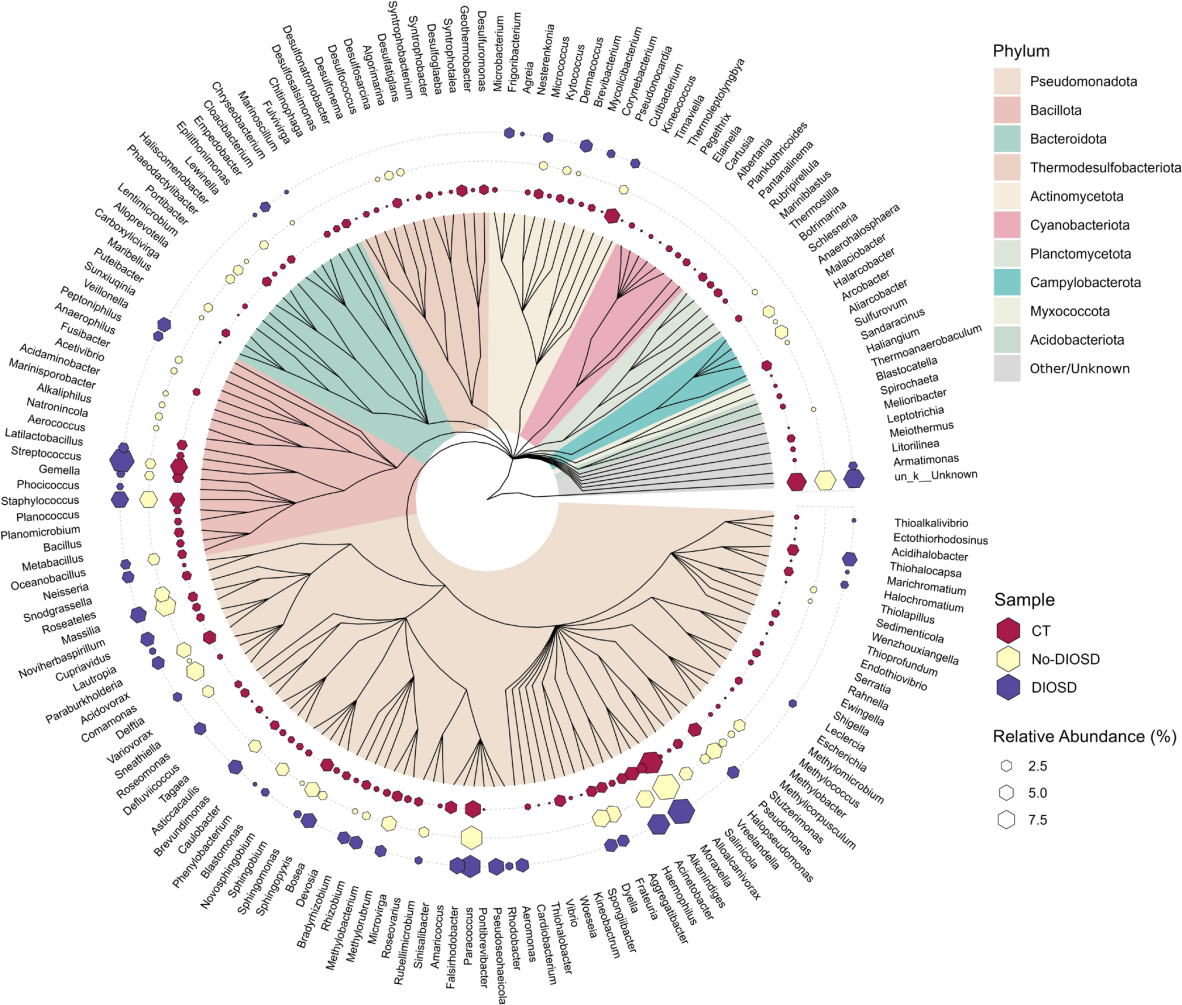
Phylogenetic tree illustrating the relative abundance of the 174 identified genera. From inside outwards: Phylogenetic tree highlighted by phylum; colored hexagons showing the genus‐level relative abundance for normal subjects (CT), dupilumab‐treated atopic dermatitis patients without ocular side effects (No‐DIOSD), and dupilumab‐treated patients with ocular side effects (DIOSD); genus classification of identified OTUs.

## Author Contributions

Conception and Design: A.L., U.R. Analysis and interpretation: A.L., R.F., F.C., J.O., U.R. Writing the article: A.L., U.R., J.O. Critical revision of the article: J.O. Final approval of the article: all authors. Data Collection: A.L., U.R., R.F., F.C. Provision of materials, patients, or resources: A.L., U.R. Statistical expertise: F.C. Obtaining funding: U.R. Literature search: A.L., U.R. Administrative, technical, or logistic support: A.L., R.F., U.R., F.C.

## Conflicts of Interest

Andrea Leonardi: Consultancy for Bausch and Lomb, Dompè, FAES Farma, FIDIA, Santen, Théa, SIFI. The other authors have no conflicts of interest.

## Supporting information


**Table S1:** all70104‐sup‐0001‐TablesS1‐S3.docx.

## Data Availability

The data that support the findings of this study are available from the corresponding author upon reasonable request.

## References

[1] R. Achten , J. Thijs , M. van der Wal , et al., “Dupilumab‐Associated Ocular Surface Disease in Atopic Dermatitis Patients: Clinical Characteristics, Ophthalmic Treatment Response and Conjunctival Goblet Cell Analysis,” Allergy 78, no. 8 (2023): 2266–2276. 36934403 10.1111/all.15717

[2] V. Patra , N. Woltsche , N. Bordag , et al., “Metabolomic and Lipidomic Alterations in Patients With Atopic Dermatitis With Dupilumab‐Associated Ocular Surface Disease,” JID Innovations 5, no. 3 (2025): 100361. 40242789 10.1016/j.xjidi.2025.100361 PMC12002936

[3] K. Thormann , A. S. Luthi , F. Deniau , et al., “Dupilumab‐Associated Ocular Surface Disease Is Characterized by a Shift From Th2/Th17 Toward Th1/Th17 Inflammation,” Allergy 79, no. 4 (2024): 937–948. 38317432 10.1111/all.16045

[4] J. Ozkan , S. Nielsen , C. Diez‐Vives , M. Coroneo , T. Thomas , and M. Willcox , “Temporal Stability and Composition of the Ocular Surface Microbiome,” Scientific Reports 7, no. 1 (2017): 9880. 28852195 10.1038/s41598-017-10494-9 PMC5575025

[5] J. Andersson , J. K. Vogt , M. D. Dalgaard , O. Pedersen , K. Holmgaard , and S. Heegaard , “Ocular Surface Microbiota in Patients With Aqueous Tear‐Deficient Dry Eye,” Ocular Surface 19 (2021): 210–217. 32931939 10.1016/j.jtos.2020.09.003

[6] P. Aragona , C. Baudouin , J. M. Benitez Del Castillo , et al., “The Ocular Microbiome and Microbiota and Their Effects on Ocular Surface Pathophysiology and Disorders,” Survey of Ophthalmology 66, no. 6 (2021): 907–925. 33819460 10.1016/j.survophthal.2021.03.010

