# Supplementary material for: Ocular Microbiome in Dupilumab‐Induced Ocular Surface Disease
Source: Allergy. 2025 Oct 16;81(3):917–9. doi: 10.1111/all.70104 (PMC12954552; doi:10.1111/all.70104)
Supplement: Supplementary file 1 — Table S1: all70104‐sup‐0001‐TablesS1‐S3.docx. [file ALL-81-917-s001.docx]

**Ocular microbiome in dupilumab-induced ocular surface disease (DIOSD)**

Andrea Leonardi, Riccardo Frizzo, Fabiano Cavarzeran, Jerome Ozkan, Umberto Rosani

**Supplementary Tables**

**Supplementary Table 1.** Clinical data of dupilumab-treated patients without ocular side effects (No-DIOSD), and with ocular surface disease (DIOSD).

|  | Atopic dermatitis patients with  ocular surface disease (DIOSD) (N=8) | | |  | Atopic dermatitis patients without ocular surface disease (No-DIOSD) (N=5) | |  |
| --- | --- | --- | --- | --- | --- | --- | --- |
| *Variable* | | *Mean (SD; Range)* | *Median (IQR)* |  | *Mean (SD; Range)* | *Median (IQR)* | *P-value** |
| Lid Eczema | | 1.86 (0.90; 1 – 3) | 2.0 (1 – 3) |  | 0.20 (0.45; 0 – 1) | 0.0 (0 – 0) | **0.0088** |
| Blepharitis | | 1.86 (0.90; 1 – 3) | 2.0 (1 – 3) |  | 0.80 (0.84; 0 – 2) | 1.0 (0 – 1) | 0.0885 |
| Conjunctival hyperemia | | 2.14 (0.38; 2 – 3) | 2.0 (2 – 2) |  | 0.20 (0.45; 0 – 1) | 0.0 (0 – 0) | **0.0026** |
| Limbal inflammation | | 2.00 (1.29; 0 – 4) | 2.0 (1 – 3) |  | 0.00 (0.00; 0 – 0) | 0.0 (0 – 0) | **0.0113** |
| Corneal epitheliopathy | | 0.43 (0.53; 0 – 1) | 0.0 (0 – 1) |  | 0.20 (0.45; 0 – 1) | 0.0 (0 – 0) | 0.4879 |
| Conjunctival fibrosis | | 0.57 (0.79; 0 – 2) | 0.0 (0 – 1) |  | 0.00 (0.00; 0 – 0) | 0.0 (0 – 0) | 0.1345 |
| Total symptoms score (VAS) | | 7.71 (1.80; 6 – 10) | 8.0 (6 – 10) |  | 1.20 (1.30; 0 – 3) | 1.0 (0 – 2) | **0.0052** |
| AD severity score at baseline** | | 26.4 (1.7; 24.4-29.8) | 26.3 (25.1-27.2) |  | 26.4 (1.3; 24.8-28.1) | 26.1 (25.5-27.3) | 1.0000 |
| Duration from DIOSD onset (weeks) | | 15.1 (8.9; 3 – 26) | 16 (7.5 – 22.5) |  |  |  |  |
| Duration from DIOSD onset to conjunctival swab collection (weeks) | | 7.6 (4.7; 2 – 13) | 8 (3.5 – 12) |  |  |  |  |

Ocular signs are reported using a 0-4 scale (0= none; 4= very severe)

Total symptoms score is reported as a subjective VAS scale 0-10

* Wilcoxon Sum Rank Test with Normal approximation and a continuity correction of 0.5

**Eczema Area and Severity Index (EASI)

**Supplementary Table 2.** Relative abundance in the 3 groups of patients at the phylum level. The entries are ordered by the difference of abundance between DIOSD and control. The difference between No-DIOSD and control is also reported.

| **Phylum** | **CT** | **DIOSD** | **No-DIOSD** | **Difference (DIOSD-CT)** | **Difference (No-DIOSD-CT)** |
| --- | --- | --- | --- | --- | --- |
| Pseudomonadota | 53.94 | 67.33 | 69.64 | 13.40 | 15.71 |
| Bacillota | 13.69 | 18.65 | 10.50 | 4.96 | -3.19 |
| Armatimonadota | 0.00 | 0.84 | 0.00 | 0.84 | 0.00 |
| Unknown | 4.66 | 5.13 | 5.67 | 0.47 | 1.01 |
| Spirochaetota | 0.00 | 0.00 | 0.23 | 0.00 | 0.23 |
| Ignavibacteriota | 0.13 | 0.00 | 0.00 | -0.13 | -0.13 |
| Chloroflexota | 0.18 | 0.00 | 0.00 | -0.18 | -0.18 |
| Deinococcota | 0.29 | 0.00 | 0.00 | -0.29 | -0.29 |
| Myxococcota | 0.31 | 0.00 | 0.00 | -0.31 | -0.31 |
| Acidobacteriota | 0.64 | 0.00 | 0.00 | -0.64 | -0.64 |
| Actinomycetota | 7.10 | 6.43 | 2.80 | -0.67 | -4.31 |
| Fusobacteriota | 0.71 | 0.00 | 0.00 | -0.71 | -0.71 |
| Campylobacterota | 1.22 | 0.00 | 4.41 | -1.22 | 3.19 |
| Cyanobacteriota | 2.42 | 0.00 | 0.00 | -2.42 | -2.42 |
| Planctomycetota | 2.99 | 0.00 | 0.33 | -2.99 | -2.66 |
| Bacteroidota | 5.50 | 1.62 | 4.57 | -3.88 | -0.93 |
| Thermodesulfobacteriota | 6.20 | 0.00 | 1.83 | -6.20 | -4.36 |

**Supplementary Table 3.** Relative abundance in the 3 groups of patients at the genus level. The entries are ordered by the difference of abundance between DIOSD and control. The difference between No-DIOSD and control is also reported.

| **Genus** | **CT** | **DIOSD** | **No-DIOSD** | **DIOSD - CT** | **No-DIOSD - CT** |
| --- | --- | --- | --- | --- | --- |
| Streptococcus | 3.52 | 7.77 | 1.22 | 4.25 | -2.30 |
| Acinetobacter | 2.41 | 6.14 | 4.11 | 3.74 | 1.71 |
| Pseudoseohaeicola | 0.00 | 3.42 | 0.00 | 3.42 | 0.00 |
| Falsirhodobacter | 0.00 | 3.27 | 0.00 | 3.27 | 0.00 |
| Paracoccus | 3.53 | 6.26 | 6.87 | 2.73 | 3.34 |
| Sphingopyxis | 0.41 | 2.98 | 0.91 | 2.57 | 0.50 |
| Moraxella | 6.88 | 9.34 | 9.54 | 2.47 | 2.66 |
| Veillonella | 0.00 | 2.41 | 0.00 | 2.41 | 0.00 |
| Aeromonas | 0.00 | 2.22 | 0.00 | 2.22 | 0.00 |
| Massilia | 1.07 | 3.23 | 5.60 | 2.16 | 4.53 |
| Rhizobium | 0.32 | 2.16 | 0.74 | 1.84 | 0.42 |
| Cupriavidus | 0.74 | 2.46 | 0.00 | 1.72 | -0.74 |
| Brevundimonas | 0.97 | 2.56 | 1.92 | 1.60 | 0.95 |
| Brevibacterium | 0.39 | 1.95 | 0.23 | 1.56 | -0.16 |
| Neisseria | 0.18 | 1.71 | 0.00 | 1.52 | -0.18 |
| Frigoribacterium | 0.00 | 1.28 | 0.00 | 1.28 | 0.00 |
| Empedobacter | 0.00 | 1.22 | 0.00 | 1.22 | 0.00 |
| Peptoniphilus | 0.00 | 1.15 | 0.00 | 1.15 | 0.00 |
| Thiohalocapsa | 1.57 | 2.61 | 0.00 | 1.05 | -1.57 |
| Staphylococcus | 2.72 | 3.73 | 3.88 | 1.01 | 1.16 |
| Roseomonas | 0.88 | 1.82 | 0.00 | 0.94 | -0.88 |
| Delftia | 0.00 | 0.93 | 0.00 | 0.93 | 0.00 |
| Bradyrhizobium | 0.98 | 1.90 | 0.33 | 0.92 | -0.65 |
| Armatimonas | 0.00 | 0.84 | 0.00 | 0.84 | 0.00 |
| Leclercia | 0.00 | 0.67 | 0.00 | 0.67 | 0.00 |
| Dyella | 1.42 | 2.08 | 4.05 | 0.65 | 2.63 |
| Rhodobacter | 0.00 | 0.64 | 0.00 | 0.64 | 0.00 |
| Rubellimicrobium | 0.00 | 0.64 | 1.26 | 0.64 | 1.26 |
| Latilactobacillus | 0.61 | 1.20 | 0.00 | 0.59 | -0.61 |
| Phocicoccus | 0.00 | 0.50 | 0.00 | 0.50 | 0.00 |
| Lautropia | 0.00 | 0.50 | 0.00 | 0.50 | 0.00 |
| Unknown | 4.66 | 5.13 | 5.67 | 0.47 | 1.01 |
| Frateuria | 1.38 | 1.71 | 3.68 | 0.33 | 2.30 |
| Methylorubrum | 1.33 | 1.60 | 2.85 | 0.27 | 1.52 |
| Blastomonas | 0.52 | 0.76 | 2.14 | 0.24 | 1.61 |
| Oceanobacillus | 0.91 | 1.15 | 1.66 | 0.23 | 0.74 |
| Agreia | 0.00 | 0.20 | 0.00 | 0.20 | 0.00 |
| Chryseobacterium | 0.00 | 0.20 | 0.00 | 0.20 | 0.00 |
| Corynebacterium | 0.60 | 0.76 | 0.00 | 0.16 | -0.60 |
| Micrococcus | 1.11 | 1.18 | 0.81 | 0.07 | -0.29 |
| Marichromatium | 0.13 | 0.20 | 0.00 | 0.07 | -0.13 |
| Anaerophilus | 0.00 | 0.00 | 0.62 | 0.00 | 0.62 |
| Fusibacter | 0.00 | 0.00 | 0.47 | 0.00 | 0.47 |
| Acidaminobacter | 0.00 | 0.00 | 0.33 | 0.00 | 0.33 |
| Alkaliphilus | 0.00 | 0.00 | 0.62 | 0.00 | 0.62 |
| Natronincola | 0.00 | 0.00 | 0.33 | 0.00 | 0.33 |
| Marinisporobacter | 0.00 | 0.00 | 0.41 | 0.00 | 0.41 |
| Lentimicrobium | 0.00 | 0.00 | 0.97 | 0.00 | 0.97 |
| Maribellus | 0.00 | 0.00 | 0.88 | 0.00 | 0.88 |
| Cloacibacterium | 0.00 | 0.00 | 0.23 | 0.00 | 0.23 |
| Portibacter | 0.00 | 0.00 | 1.15 | 0.00 | 1.15 |
| Aliarcobacter | 0.00 | 0.00 | 0.23 | 0.00 | 0.23 |
| Arcobacter | 0.00 | 0.00 | 2.22 | 0.00 | 2.22 |
| Halarcobacter | 0.00 | 0.00 | 0.23 | 0.00 | 0.23 |
| Malaciobacter | 0.00 | 0.00 | 1.72 | 0.00 | 1.72 |
| Schlesneria | 0.00 | 0.00 | 0.33 | 0.00 | 0.33 |
| Sphingobium | 0.00 | 0.00 | 0.23 | 0.00 | 0.23 |
| Acidovorax | 0.00 | 0.00 | 0.23 | 0.00 | 0.23 |
| Variovorax | 0.00 | 0.00 | 1.71 | 0.00 | 1.71 |
| Roseateles | 0.00 | 0.00 | 2.87 | 0.00 | 2.87 |
| Sedimenticola | 0.00 | 0.00 | 0.23 | 0.00 | 0.23 |
| Halopseudomonas | 0.00 | 0.00 | 1.12 | 0.00 | 1.12 |
| Stutzerimonas | 0.00 | 0.00 | 0.74 | 0.00 | 0.74 |
| Spirochaeta | 0.00 | 0.00 | 0.23 | 0.00 | 0.23 |
| Thioalkalivibrio | 0.26 | 0.20 | 0.00 | -0.06 | -0.26 |
| Paraburkholderia | 2.09 | 2.00 | 2.84 | -0.09 | 0.75 |
| Kineococcus | 0.13 | 0.00 | 0.00 | -0.13 | -0.13 |
| Pseudonocardia | 0.13 | 0.00 | 0.00 | -0.13 | -0.13 |
| Carboxylicivirga | 0.13 | 0.00 | 0.00 | -0.13 | -0.13 |
| Sunxiuqinia | 0.13 | 0.00 | 0.00 | -0.13 | -0.13 |
| Pantanalinema | 0.13 | 0.00 | 0.00 | -0.13 | -0.13 |
| Elainella | 0.13 | 0.00 | 0.00 | -0.13 | -0.13 |
| Thermoleptolyngbya | 0.13 | 0.00 | 0.00 | -0.13 | -0.13 |
| Melioribacter | 0.13 | 0.00 | 0.00 | -0.13 | -0.13 |
| Sandaracinus | 0.13 | 0.00 | 0.00 | -0.13 | -0.13 |
| Asticcacaulis | 0.13 | 0.00 | 0.00 | -0.13 | -0.13 |
| Pontibrevibacter | 0.13 | 0.00 | 0.00 | -0.13 | -0.13 |
| Defluviicoccus | 0.13 | 0.00 | 0.00 | -0.13 | -0.13 |
| Spongiibacter | 0.13 | 0.00 | 0.00 | -0.13 | -0.13 |
| Acidihalobacter | 0.13 | 0.00 | 0.00 | -0.13 | -0.13 |
| Ectothiorhodosinus | 0.13 | 0.00 | 0.00 | -0.13 | -0.13 |
| Thiolapillus | 0.13 | 0.00 | 0.47 | -0.13 | 0.34 |
| Wenzhouxiangella | 0.13 | 0.00 | 0.00 | -0.13 | -0.13 |
| Serratia | 0.13 | 0.00 | 0.00 | -0.13 | -0.13 |
| Methylococcus | 0.13 | 0.00 | 0.00 | -0.13 | -0.13 |
| Thiohalobacter | 0.13 | 0.00 | 0.00 | -0.13 | -0.13 |
| Vibrio | 0.13 | 0.00 | 0.00 | -0.13 | -0.13 |
| Algorimarina | 0.13 | 0.00 | 0.66 | -0.13 | 0.53 |
| Desulfococcus | 0.13 | 0.00 | 0.23 | -0.13 | 0.10 |
| Desulfosalsimonas | 0.13 | 0.00 | 0.00 | -0.13 | -0.13 |
| Litorilinea | 0.18 | 0.00 | 0.00 | -0.18 | -0.18 |
| Haliangium | 0.18 | 0.00 | 0.00 | -0.18 | -0.18 |
| Kineobactrum | 0.18 | 0.00 | 0.00 | -0.18 | -0.18 |
| Rahnella | 0.18 | 0.00 | 0.00 | -0.18 | -0.18 |
| Methylomicrobium | 0.18 | 0.00 | 0.00 | -0.18 | -0.18 |
| Salinicola | 0.18 | 0.00 | 2.22 | -0.18 | 2.04 |
| Geothermobacter | 0.18 | 0.00 | 0.00 | -0.18 | -0.18 |
| Desulfoglaeba | 0.18 | 0.00 | 0.00 | -0.18 | -0.18 |
| Syntrophobacter | 0.18 | 0.00 | 0.00 | -0.18 | -0.18 |
| Kytococcus | 0.23 | 0.00 | 0.00 | -0.23 | -0.23 |
| Pegethrix | 0.23 | 0.00 | 0.00 | -0.23 | -0.23 |
| Sinisalibacter | 0.23 | 0.00 | 0.00 | -0.23 | -0.23 |
| Methylicorpusculum | 0.23 | 0.00 | 0.52 | -0.23 | 0.30 |
| Methylobacter | 0.23 | 0.00 | 1.69 | -0.23 | 1.46 |
| Alloalcanivorax | 0.23 | 0.00 | 0.00 | -0.23 | -0.23 |
| Mycolicibacterium | 0.26 | 0.00 | 0.00 | -0.26 | -0.26 |
| Haliscomenobacter | 0.26 | 0.00 | 0.00 | -0.26 | -0.26 |
| Botrimarina | 0.26 | 0.00 | 0.00 | -0.26 | -0.26 |
| Endothiovibrio | 0.26 | 0.00 | 0.00 | -0.26 | -0.26 |
| Phenylobacterium | 0.47 | 0.20 | 0.00 | -0.27 | -0.47 |
| Microbacterium | 0.29 | 0.00 | 0.00 | -0.29 | -0.29 |
| Alloprevotella | 0.29 | 0.00 | 0.00 | -0.29 | -0.29 |
| Meiothermus | 0.29 | 0.00 | 0.00 | -0.29 | -0.29 |
| Devosia | 0.29 | 0.00 | 0.00 | -0.29 | -0.29 |
| Desulfonema | 0.29 | 0.00 | 0.00 | -0.29 | -0.29 |
| Halochromatium | 0.98 | 0.67 | 0.00 | -0.30 | -0.98 |
| Blastocatella | 0.32 | 0.00 | 0.00 | -0.32 | -0.32 |
| Thermoanaerobaculum | 0.32 | 0.00 | 0.00 | -0.32 | -0.32 |
| Nesterenkonia | 0.32 | 0.00 | 0.00 | -0.32 | -0.32 |
| Lewinella | 0.32 | 0.00 | 0.88 | -0.32 | 0.56 |
| Comamonas | 0.32 | 0.00 | 4.44 | -0.32 | 4.12 |
| Shigella | 0.32 | 0.00 | 0.00 | -0.32 | -0.32 |
| Desulfatiglans | 0.32 | 0.00 | 0.00 | -0.32 | -0.32 |
| Albertania | 0.35 | 0.00 | 0.00 | -0.35 | -0.35 |
| Timaviella | 0.37 | 0.00 | 0.00 | -0.37 | -0.37 |
| Cardiobacterium | 0.41 | 0.00 | 0.00 | -0.41 | -0.41 |
| Desulfonatronobacter | 0.43 | 0.00 | 0.00 | -0.43 | -0.43 |
| Marinoscillum | 0.45 | 0.00 | 0.00 | -0.45 | -0.45 |
| Cartusia | 0.45 | 0.00 | 0.00 | -0.45 | -0.45 |
| Caulobacter | 0.45 | 0.00 | 0.00 | -0.45 | -0.45 |
| Aggregatibacter | 0.47 | 0.00 | 0.00 | -0.47 | -0.47 |
| Pseudomonas | 2.27 | 1.79 | 3.41 | -0.48 | 1.14 |
| Novosphingobium | 0.49 | 0.00 | 0.00 | -0.49 | -0.49 |
| Syntrophobacterium | 0.49 | 0.00 | 0.00 | -0.49 | -0.49 |
| Epilithonimonas | 0.70 | 0.20 | 0.00 | -0.50 | -0.70 |
| Fulvivirga | 0.51 | 0.00 | 0.00 | -0.51 | -0.51 |
| Anaerohalosphaera | 0.51 | 0.00 | 0.00 | -0.51 | -0.51 |
| Mariniblastus | 0.54 | 0.00 | 0.00 | -0.54 | -0.54 |
| Gemella | 1.31 | 0.76 | 0.97 | -0.55 | -0.34 |
| Tagaea | 0.55 | 0.00 | 0.00 | -0.55 | -0.55 |
| Planomicrobium | 0.57 | 0.00 | 0.00 | -0.57 | -0.57 |
| Microvirga | 0.57 | 0.00 | 0.00 | -0.57 | -0.57 |
| Planococcus | 0.58 | 0.00 | 0.00 | -0.58 | -0.58 |
| Sneathiella | 0.58 | 0.00 | 0.00 | -0.58 | -0.58 |
| Acetivibrio | 0.61 | 0.00 | 0.00 | -0.61 | -0.61 |
| Methylobacterium | 0.61 | 0.00 | 0.00 | -0.61 | -0.61 |
| Planktothricoides | 0.64 | 0.00 | 0.00 | -0.64 | -0.64 |
| Metabacillus | 0.65 | 0.00 | 0.00 | -0.65 | -0.65 |
| Escherichia | 0.65 | 0.00 | 0.00 | -0.65 | -0.65 |
| Dermacoccus | 0.71 | 0.00 | 0.81 | -0.71 | 0.10 |
| Leptotrichia | 0.71 | 0.00 | 0.00 | -0.71 | -0.71 |
| Thioprofundum | 0.71 | 0.00 | 0.00 | -0.71 | -0.71 |
| Puteibacter | 0.75 | 0.00 | 0.23 | -0.75 | -0.51 |
| Noviherbaspirillum | 0.75 | 0.00 | 0.00 | -0.75 | -0.75 |
| Ewingella | 0.77 | 0.00 | 0.00 | -0.77 | -0.77 |
| Bosea | 0.78 | 0.00 | 0.00 | -0.78 | -0.78 |
| Thermostilla | 0.81 | 0.00 | 0.00 | -0.81 | -0.81 |
| Roseovarius | 0.86 | 0.00 | 0.00 | -0.86 | -0.86 |
| Rubripirellula | 0.87 | 0.00 | 0.00 | -0.87 | -0.87 |
| Chitinophaga | 0.94 | 0.00 | 0.00 | -0.94 | -0.94 |
| Snodgrassella | 0.98 | 0.00 | 0.00 | -0.98 | -0.98 |
| Phaeodactylibacter | 1.02 | 0.00 | 0.23 | -1.02 | -0.78 |
| Bacillus | 1.04 | 0.00 | 0.00 | -1.04 | -1.04 |
| Desulfosarcina | 1.09 | 0.00 | 0.94 | -1.09 | -0.15 |
| Desulfuromonas | 1.10 | 0.00 | 0.00 | -1.10 | -1.10 |
| Vreelandella | 1.11 | 0.00 | 0.00 | -1.11 | -1.11 |
| Aerococcus | 1.17 | 0.00 | 0.00 | -1.17 | -1.17 |
| Alkanindiges | 1.20 | 0.00 | 0.00 | -1.20 | -1.20 |
| Sulfurovum | 1.22 | 0.00 | 0.00 | -1.22 | -1.22 |
| Haemophilus | 1.32 | 0.00 | 0.00 | -1.32 | -1.32 |
| Sphingomonas | 2.03 | 0.67 | 2.90 | -1.36 | 0.87 |
| Syntrophotalea | 1.53 | 0.00 | 0.00 | -1.53 | -1.53 |
| Woeseia | 1.62 | 0.00 | 0.00 | -1.62 | -1.62 |
| Amaricoccus | 1.84 | 0.00 | 0.00 | -1.84 | -1.84 |
| Cutibacterium | 2.93 | 1.05 | 0.94 | -1.88 | -2.00 |
